# Prevalence and correlates of the composite index of anthropometric failure among children under 5 years old in Bangladesh

**DOI:** 10.1111/mcn.12930

**Published:** 2019-12-22

**Authors:** Md. Saimul Islam, Tuhin Biswas

**Affiliations:** ^1^ Department of Statistics University of Rajshahi Rajshahi 6205 Bangladesh; ^2^ Institute for Social Science Research University of Queensland Brisbane 4068 Australia

**Keywords:** Bangladesh, children, CIAF, underweight, stunting, wasting

## Abstract

The prevalence of stunting, wasting, and underweight are reported separately. However, the data of the multiple anthropometric failures combinations of these conventional indicators are scant. This study attempted to estimate the overall burden of undernutrition among children under 5 years old, using the composite index of anthropometric failure (CIAF), and to explore the correlates. The study used secondary data from the Bangladesh demographic and health surveys (BDHS), undertaken in 2014. CIAF provides an overall prevalence of undernutrition, which gives six mutually exclusive anthropometric measurements of height‐for‐ age, height‐for‐weight, and weight‐for‐age. Multivariable logistic regression was used to explore the correlates of CIAF. The overall prevalence of undernutrition using the CIAF was 48.3% (95% CI [47.1%, 49.5%]) among the children under 5 years old. The prevalence of anthropometric failure due to a combination of both stunting and underweight was 18.2%, wasting and underweight was 5.5%, and wasting, underweight, and stunting was 5.7%. The odds of CIAF were higher among young maternal age, having the poorest socio‐economic status, living in rural areas, higher order of birth, and received no vaccination compared with other counterparts. In Bangladesh, one out of two children has undernutrition, which is preventing the potential of the millions of children. Mothers who gave birth before age 20 living in the rural areas with belonging to lower socio‐economic status and whose children had a higher order of birth and receive no vaccination were observed as the main determinants of undernutrition. Nutrition sensitive interventions along with social protection programmes are crucial to deal the underlying causes of undernutrition.

**Key messages**

- Nearly half of the children have some form of anthropometric failures, which is a big problem.
- There were considerable geographical variations of undernutrition between urban and rural areas across all the administrative division.
- The prevalence of undernutrition among children under 5 years is significantly higher for maternal child marriage, living in poorest socio‐economic groups with belonging to rural areas, no vaccination received, and higher order of birth.

**Abbreviations**
ANC: antenatal care
BDHS: Bangladesh demography and health survey
CIAF: composite index of anthropometric failure
WHO: World Health Organization

## 1 INTRODUCTION

Child malnutrition, particularly undernutrition, remains one of the biggest health problems for developing countries. Recent global estimates reported that 45% of child deaths annually attribute to various forms of undernutrition (Black et al., 2013). More than 90% of the children who live in the African and Asian countries are stunted, and 70% are wasted, and these children are at substantial risk of acute malnutrition and death (De Onis, Brown, Blossner, & Borghi, 2012). Evidence showed that rapid economic growth may have a relationship for the reduction of undernutrition (Singh, 2014). In the past decade, Bangladesh has achieved substantial economic progress, and the gross domestic product growth rate is 7.1% per annum (Bank, 2016). Even with the significant improvement in the health sectors, the country is going through epidemiological transition from communicable disease to noncommunicable disease and nutritional transition of over nutrition to undernutrition. The level of undernutrition is still higher compared with other developing countries (Mascie‐Taylor, 2012).

According to the Bangladesh demography and health survey (BDHS) 2011, the prevalence of stunting, wasting, and underweight is still high among Bangladeshi children under 5 years old, particularly among the older age groups and living in the rural setting. Other predictors are lower socio‐economic status and lower education of parents (Chowdhury et al., 2016; National Institute of Population Research and Training [NIPORT], 2013). Such estimate of undernutrition by the conventional indicators may overlap, which would not give a comprehensive estimate of undernutrition for any country (Nandy, Irving, Gordon, Subramanian, & Smith, 2005). However, a systematic review conducted on the developing countries reported that many children have multiple anthropometric failures, which leads to a heightened risk of morbidity and mortality. Children with three combined anthropometric failures have a 12‐fold elevated risk of mortality (McDonald et al., 2013). Studies in Asian countries have found a concurrent relationship between stunting and wasting with compare with a standard population (Richard et al., 2012). A review study suggests that underweight children will experience stunting and/or wasting and some children might simultaneously experience all three forms of anthropometric failures (Achadi et al., 2016). As a result, none of these conventional nutritional indicators can really estimate the overall burden and the joint estimate of undernutrition among children under 5 years old.

A joint estimate of anthropometric failure is crucial to understand the real burden of undernutrition for any low‐ and middle‐income countries like Bangladesh. In 2000, Peter Svedberg developed a composite index of anthropometric failure (CIAF), which gives six different measurements of undernutrition using the conventional nutritional indicators, and the aggregated values of these indicators give the overall burden of undernutrition. The estimate of CIAF is helpful for modifying the existing intervention or developing a new nutritional programme with targeting specific populations.

To achieve the sustainable development goal of improved nutrition by 2030, a comprehensive estimate of undernutrition is essential for scaling up the nutritional programme. However, both the overall burden and the joint estimate of undernutrition are still absent among the under 5‐year‐old children in Bangladesh. This study utilized the dataset of the most recent BDHS 2014 to estimate the burden of undernutrition using CIAF and to identify significant covariates.

## 2 METHODS AND MATERIALS

### 2.1 Study settings

We used the BDHS dataset for this study. The survey used a sampling frame from the list of enumeration areas (EAs) of the 2011 population and housing census of the People's Republic of Bangladesh, provided by the Bangladesh Bureau of Statistics. The primary sampling unit for the survey is an EA created to have an average of about 120 households. The study is based on a two‐stage stratified sample of households. In the first stage, 600 EAs were selected with probability proportional to the EA size, with 207 EAs in urban areas and 393 in rural areas (NIPORT, 2013). A household listing was completed in each of the primary sampling unit (PSU), and then 30 households were selected from each PSU by the systematic random sampling procedures. The study population was mothers aged 15–49 years who have children aged 0–59 months. If the mother had more than one child at the same age group, then one child was randomly selected in this study (Figure S1).

### 2.2 Outcomes

The outcome variable of this study was the nutritional status among children under 5 years measured using CIAF. Svedberg had recommended six subgroups of anthropometric failure (A to F; Table 1). However, Nandy, Irving, Gordon, Subramanian, and Smith (2005) identified that children who are only underweight but are not stunted or wasted (Group—Y). Children nutritional indicators were categorized into seven groups: (A) no failure; (B) wasting only; (C) wasting and underweight; (D) wasting, stunting, and underweight; (E) stunting and underweight; (F) stunting only; and (Y) underweight only. A child is considered as undernourished, as measured in CIAF, if he or she is suffering from any anthropometric failure (B–Y) described above (Table 1).

**Table 1:** Classification of composite index of anthropometric failure to asses undernutrition among the children under 5 years

Group	Descriptions	Description of the levels	Wasting	Stunting	Underweight
A	No failure	Normal WAZ, HAZ, and WHZ	No	No	No
B	Wasting only	WAZ <−2SD but normal HAZ and WHZ	Yes	No	No
C	Wasting and underweight	WAZ and WHZ <−2 SD but HAZ normal	Yes	No	Yes
D	Stunting, wasting, and underweight	HAZ and WAZ and WHZ <−2SD	Yes	Yes	Yes
E	Stunting and underweight	HAZ and WHZ<−2SD but WAZ normal	No	Yes	Yes
F	Stunting only	HAZ<−2SD but normal WAZ and WHZ	No	Yes	No
Y^a^	Underweight only	WHZ<−2SD but normal HAZ and WAZ	No	No	Yes

Based on Nandy et al.

### 2.3 Covariates

A series of information was extracted from the BDHS including sociodemographic and economic characteristics (child's age, child's sex, birth order, preceding birth interval, antenatal care [ANC] visit, maternal marital status, maternal educational status, maternal age at first birth, size of household, place of residence, and wealth index). The preceding birth interval was categorized as first birth, <24 months, 24–47 months, and ≥48 months. The current marital status was categorized into formerly married includes divorce, widowed, and currently married. ANC visit was categorized by less than four ANC visits and equal or more than four ANC visits received. The BDHS survey collected data from household ownership of assets and consumer goods such as the source of drinking water, type of toilet facilities, type of fuel, ownership of various durable goods, and other characteristics relating to socio‐economic status of the household. The household wealth index is an asset‐based socio‐economic index constructed using principal component analysis and following standard guidelines(“Wealth index construction,” the DHS programme). For this analysis, the wealth index was grouped into five categories: poorest, poor, middle, rich, and richest.

### 2.4 Measurement

The 2014 BDHS collected anthropometric data by measuring the height and weight of all children under age 5 in the selected households (NIPORT, 2013). The nutritional status of children in the survey population is compared with the World Health Organization (WHO) Child Growth Standards, which are based on an international sample of ethnically, culturally, and genetically diverse healthy children living under optimal conditions to achieve a child's full genetic growth potential. A child who is more than two standard deviations below the median (−2 SD) of the WHO reference population in terms of height for age is considered stunted, weight for height is considered wasted, and weight for age is considered underweight (Group & de Onis, 2006).

### 2.5 ETHICS

This study has utilized secondary data obtained from the BDHS 2014 that was collected by the MEASURE DHS programme. Ethical approval has been obtained from the institutional review board of intermediate care facility of Calverton, Maryland, USA. Therefore, ethical approval was not required, since DHS provided the data for the secondary analysis research. Access to the datasets could be obtained through online registration (http://dhsprogram.com/data/Access‐Instructions.cfm).

### 2.6 DATA ANALYSIS

Prevalence of CIAF was calculated if a child has any one of the six different types of anthropometric failures out of total children under 5 years. Categorical variables were summarized using frequency distribution, and continuous variables were summarized using mean, standard deviation, and median according to the nature of data. Bivariate analysis was done by chi‐square test to assess factors associated with CIAF. *P* value less than .05 in the bivariate analysis would be considered as candidates to be included in a multivariable regression model. Multivariable logistic regression model, enter method was applied to report the unadjusted and adjusted odds ratio and 95% confidence interval, and statistical significance was considered with a *P* < .05. All the analyses were performed after adjustment with the cluster and sampling weight. All missing values were excluded from the analysis. The analysis was performed by IBM SPSS v21 software.

## 3 RESULTS

### 3.1 Basic characteristics

We studied 6,965 children under 5 years old and their mothers. The average age of the children was 30 (17) months, where 51% of the children were female, and 68% lived in rural areas. Of the children, 39% was the firstborn child of their parents, and 7% of the children were born less than 24 months after the first birth. The proportion of short birth interval (<24 months) was higher in the rural than the urban areas(16% vs. 13%; *P* = .005). Among the children, 23% were born through caesarean section. This proportion of caesarean birth was higher in the urban than rural areas (36% vs. 18%, *P* < .001). Among the children, 20% had a low birthweight. Approximately 31% of the children were third or more in the order of birth. Four percent of the children had acute respiratory infraction, 5% was suffering from diarrhoea, and 37% had a fever before 2 weeks of the survey (Table 2).

**Table 2:** Basic characteristics of the study participants

Variables	Label	Total = 6,965 (%)
Child information		
Sex	Male	3,571 (51)
	Female	3,394 (49)
Age, Mean (SD) (month)		30(17)
	0–11	1,344 (19)
	12–23	1,456 (21)
	24–35	1,406 (20)
	36–47	1,376 (20)
	48–59	1,383 (20)
Birth interval in month		
	First birth	2,714 (39)
	<24	4,76 (7)
	24–47	1,388 (20)
	48+	2,387 (34)
Birth order	1st	2,700 (39)
	2nd	2,091 (30)
	3rd	1,104 (16)
	≥4th	1,070 (15)
ANC care (*n* = 4,084)		
	≥4	1,301 (32)
	<4	2,747 (68)
Diarrhoea^a^	Yes	337 (5)
	No	6,622 (95)
Fever^a^	Yes	2,569 (37)
	No	4,389 (63)
Delivery by C‐section, *n* = 4,204	Yes	982 (23)
	No	3,222 (77)
Ever had vaccination, *n* = 2,448	Yes	2,133 (87.1)
	No	315 (12.9)
Child size at birth, *n* = 4,728	Larger than average	616 (13)
	Average	3,184 (67)
	Smaller than average	928 (20)
Acute respiratory infraction (ARI), *n* = 6,899	Yes	280 (4)
	No	6,619 (96)
Mother current age, Mean (SD)		26 (6)
Mother age at birth		
	<20	5,085 (73)
	≥20	1,880 (27)
Highest educational level	No education	1,076 (15.4)
	Primary	1,934 (27.8)
	Secondary	3,219 (46.2)
	Higher	736 (10.6)
Respondent currently working	Yes	1,747 (25.1)
	No	5,217 (74.9)
Marital status	Formerly Married	83 (1.2)
	Currently Married	6,882 (98.8)
BMI, *n =* 6,946	Underweight	1,556 (22)
	Normal weight	4,067 (59)
	Overweight and obesity	1,323 (19)
Wealth index	Poorest	1,515 (22)
	Poor	1,307 (19)
	Middle	1,379 (20)
	Rich	1,420 (20)
	Richest	1,344 (19)
Place of residence	Urban	2,188 (31.4)
	Rural	4,777 (68.6)
Exposed with media	TV/Radio/Newspaper	4,315 (62)
	Not at all	2,650 (38)

Abbreviations: ANC, antenatal care; BMI, body mass index.

a Last 2 weeks.

The average age of the mothers of children was 26 (6) years, and 46% had completed secondary education. In this study, 4,084 mothers reported about ANC visit, of which 68% received less than four times ANC, and this proportion was higher in rural than urban areas (74% vs. 55%; *P* < .001). Of the mothers, 22% had underweight, and 19% had overweight and obesity. The proportion of overweight was higher among the mothers in urban areas than rural areas (29% vs. 14%; *P* < .001; Table 1).

### 3.2 Joint estimate of child undernutrition

In the study, 48% of the children have one or more forms of undernutrition including underweight and stunting (18%); stunting only (13%); wasting, underweight, and stunting(6%); wasting and underweight(6%); wasting only(3%); and underweight only (3%).Stunting only was significantly higher among male than female children (13% vs. 11%; *P* < .001). The joint prevalence of wasting and underweight was higher among younger children (0–23 months) compared with older age (24–59 months). On the other hand, the prevalence of underweight and stunting was lower among younger age children compared with the older age group. The combined prevalence of stunting, wasting, and underweight significantly varied by the age of the children (Table 3).

**Table 3:** Prevalence and 95% CI of the composite index for different form of anthropometric failure by the characteristics of children, mother, and socio‐economic positions

Variables	Total	CIAF	No failure	Wasting only	Wasting and underweight	Wasting, underweight and stunting	Underweight and stunting	Stunting only	Underweight only
Overall,		48.3 [47.1, 49.5]	51.7 [50.5, 52.8]	3.2 [2.8, 3.6]	5.5 [5.0, 6.1]	5.7 [5.2, 6.2]	18.2 [17.3, 19.2]	12.6 [11.9, 13.4]	3.0 [2.6, 3.4]
Child information									
Sex									
Male	3,571	49.2 [46.9, 51.5]	50.8 [48.5, 53.1]	3.7 [3.0, 4.6]	5.8 [4.9, 6.8]	5.6 [4.8, 6.5]	18.0 [16.2, 20.0]	13.3 [11.9, 14.9]*	2.8 [2.2, 3.5]*
Female	3,394	47.1 [44.9, 49.3]	52.9 [50.7, 55.1]	2.6 [2.1, 3.3]	5.7 [4.6, 7.1]	5.2 [4.4, 6.2]	19.0 [17.3, 20.9]	11.3 [10, 12.7]	3.2 [2.6, 3.9]
Age									
0–11 months	1,344	36.6 [33.2, 40.3]*	63.4 [59.7, 66.8] *	7.9 [6.2, 10.1] *	8.2 [6.3, 10.6] *	2.8 [1.9, 4.1] *	5.7 [4.3, 7.4] *	8.8 [6.6, 11.6] *	3.3 [2.2, 4.8]*
12–23 months	1,456	48.9 [45.4, 52.5]	51.1 [47.5, 54.6]	2.1 [1.4, 3.1]	6.8 [5.4, 8.5]	6.2 [4.8, 7.9]	17.3 [14.4, 20.7]	14.9 [12.9, 17.2]	1.7 [1.1, 2.5]
24–35 months	1,406	52.2 [48.7, 55.5]	47.8 [44.5, 51.3]	1.7 [1.1, 2.6]	5.8 [3.9, 8.5]	5.3 [4.1, 6.8]	22.1 [19.5, 24.9]	13.9 [11.7, 16.3]	3.4 [2.4, 4.8]
36–47 months	1,376	53.4 [50.1, 56.7]	46.6 [43.3, 49.9]	1.8 [1.1, 2.7]	3.3 [2.4, 4.6]	6.6 [5.3, 8.2]	24.2 [21.3, 27.5]	14.5 [12.4, 16.9]	3.0 [2.2, 4.2]
48–59 months	1,383	49.7 [46, 53.4]	50.3 [46.6, 54]	2.7 [1.9, 3.8]	4.6 [3.5, 6.2]	6.1 [4.7, 7.7]	23.4 [20.3, 26.7]	9.3 [7.5, 11.6]	3.6 [2.7, 4.8]
Preceding Birth interval									
First birth	2,714	43.7 [41.3, 46.2]*	56.3 [53.8, 58.7]	2.7 [2.1, 3.6]	6.8 [5.7, 8]*	4.6 [3.8, 5.6]*	14.9 [13.3, 16.6]	12.2 [10.6, 14]	2.6 [2, 3.4]
<24 month	476	59.3 [53.3, 65]	40.7 [35, 46.7]	3.9 [2.4, 6.3]	4.8 [2.5, 9]	7.2 [4.9, 10.5]	28.5 [22.5, 35.4]	11.6 [8.7, 15.5]	3.2 [1.7, 6]
24–47 months	1,388	55.7 [52, 59.3]	44. 3 [40.7, 48]	2.9 [2, 4.2]	6.4 [4.4, 9.3]	6.6 [5.2, 8.3]	23.2 [20.2, 26.5]	13.8 [11.6, 16.3]	2.7 [1.9, 3.8]
≥48 months	2,387	46.5 [43.9, 49.1]	53.5 [50.9, 56.1]	3.7 [2.9, 4.9]	4.4 [3.6, 5.4]	5.2 [4.3, 6.4]	17.8 [15.7, 20.2]	11.8 [10.3, 13.5]	3.5 [2.7, 4.5]
Birth order									
1st	2,700	53.5 [51, 56.1]	46.5 [43.9, 49.0]	2.6 [2.0, 3.3]	5.7 [4.5, 7.3]	5.8 [4.8, 6.9]	22.3 [20.1, 24.6]	13.3 [11.8, 15]	3.9 [3.1, 4.8]
2nd	2,091	49.1 [46.2, 51.9]	50.9 [48.1, 53.8]	3.5 [2.6, 4.8]	6.5 [5.3, 8.1]	5.1 [4.1, 6.3]	19.5 [17, 22.2]	12.4 [10.8, 14.3]	2.0 [1.4, 2.8]
3rd	1,104	46.3 [42.3, 50.4]	53.7 [49.6, 57.7]	3.1 [2.2, 4.4]	6.1 [4.5, 8.1]	5.0 [3.7, 6.6]	15.5 [13.1, 18.2]	13.1 [10.3, 16.5]	3.7 [2.5, 5.4]
≥4	1,070	34.8 [31.3, 38.5]	65.2 [61.5, 68.7]	4.2 [2.9, 6.1]	4 [2.7, 5.9]	5.5 [4, 7.6]	10.4 [8.4, 12.9]	8.8 [7, 11]	1.9 [1.1, 3.2]
ANC care (*n* = 4,084)									
≥4	1,301	49.6 [47.1, 52.1]	64 [60.2, 67.8]	3.5 [2.4, 5.2]	6.7 [4.5, 9.7]	3.5 [2.5, 4.9]	9.1 [7.3, 11.3]	10.6 [8.2, 13.5]	2.5 [1.7, 3.6]
<4	2,747	36 [32.2, 39.8]	50.4 [47.9, 52.9]	4.1 [3.3, 5.2]	7.2 [6, 8.6]	5.5 [4.6, 6.7]	16.9 [14.9, 19]	12.9 [11.5, 14.5]	2.9 [2.2, 3.9]
Diarrhoea^a^									
Yes	337	53.2 [44.7, 61.5]	52.2 [50.6, 53.8]	3.3 [2.9, 3.9]	5.8 [5, 6.6]	5.2 [4.6, 5.9]	18.5 [17.2, 19.8]	11.9 [11, 12.9]	3.1 [2.6, 3.7]
No	6,622	47.8 [46.2, 49.4]	52.2 [50.6, 53.8]	3.3 [2.9, 3.9]	5.8 [5, 6.6]	5.2 [4.6, 5.9]	18.5 [17.2, 19.8]	11.9 [11, 12.9]	3.1 [2.6, 3.7]
Fever^a^									
Yes	2,569	52.5 [49.8, 55.1]	47.5 [44.9, 50.2]	3.3 [2.6, 4.3]	6.8 [5.4, 8.6]	6.8 [5.8, 8.1]	19.1 [17.1, 21.3]	12.9 [11.2, 14.9]	3.5 [2.7, 4.5]
No	4,389	45.6 [43.7, 47.6]	54.4 [52.4, 56.3]	3.1 [2.5, 3.8]	5.1 [4.4, 6]	4.5 [3.8, 5.3]	18.2 [16.6, 19.9]	12 [10.9, 13.2]	2.7 [2.2, 3.3]
Delivery by C‐section, *n* = 4,204									
Yes	982	33.8 [29.9, 38]	66.2 [62, 70.1]	4.4 [3.2, 6.2]	7.1 [4.5, 11]	2.5 [1.6, 4]	7.3 [5.5, 9.8]	10.5 [8.4, 13]	1.9 [1.1, 3.5]
No	3,222	49.8 [47.4, 52.1]	50.2 [47.9, 52.6]	3.6 [2.9, 4.6]	6.8 [5.8, 8.1]	5.5 [4.6, 6.5]	17.5 [15.7, 19.5]	13.3 [11.8, 14.9]	3 [2.3, 3.8]
Ever had vaccination (*n* = 2,448)									
Yes	2,133	47.2 [44.2, 50.1]	52.8 [49.9, 55.8]	2.8 [2.1, 3.7]	5.2 [3.8, 7]	4.7 [3.8, 5.9]	19.4 [17.1, 21.9]	12.5 [10.8, 14.4]	2.7 [2, 3.5]
No	315	56.3 [48.6, 63.7]	43.7 [36.3, 51.4]	8.9 [5.2, 14.8]	2.4 [1.3, 4.5]	5.4 [3.3, 8.6]	23.2 [15.4, 33.2]	12.5 [8.3, 18.2]	4 [1.9, 8.6]
Child size at birth, *n* = 4,728									
Larger than average	616	47.6 [42, 53.3]	52.4 [46.7, 58]	3.9 [2.4, 6.3]	3.5 [2.2, 5.7]	5.3 [3.5, 7.9]	19.4 [15.5, 24.1]	10.9 [8.1, 14.3]	4.6 [2.9, 7.2]
Average	3,184	49.9 [47.4, 52.3]	50.1 [47.7, 52.6]	2.9 [2.2, 3.7]	5.8 [4.7, 7]	5.6 [4.7, 6.7]	19.2 [17.3, 21.2]	13.6 [12, 15.5]	2.8 [2.2, 3.6]
Smaller than average	928	42.6 [37.8, 47.4]	57.4 [52.6, 62.2]	3.1 [1.9, 5.1]	5 [3.4, 7.3]	4.9 [3.5, 6.9]	15.8 [12.2, 20.2]	10.7 [8.5, 13.5]	3 [1.9, 4.7]
ARI, *n* = 6,899									
Yes	280	54.4 [45.8, 62.7]	45.6 [37.3, 54.2]	6.6 [3.8, 11]	3.6 [2, 6.5]	6.7 [4.1, 10.6]	20 [14.6, 26.7]	14.2 [9.9, 20]	3.3 [1.5, 7.2]
No	6,619	47.8 [46.1, 49.4]	52.2 [50.6, 53.9]	3 [2.6, 3.6]	5.8 [5.1, 6.7]	5.3 [4.7, 6]	18.3 [17, 19.7]	12.3 [11.3, 13.3]	3 [2.5, 3.5]
Maternal information									
Age at first birth									
<20	5,085	50.4 [48.5, 52.2]	49.6 [47.8, 51.5]	3.2 [2.7, 3.9]	5.5 [4.7, 6.3]	5.9 [5.2, 6.7]	19.9 [18.4, 21.5]	12.9 [11.7, 14.2]	2.9 [2.4, 3.5]
≥20	1,880	42.2 [39.2, 45.2]	57.8 [54.8, 60.8]	3.1 [2.4, 4.1]	6.5 [4.8, 8.8]	3.9 [3, 5.1]	14.7 [12.6, 17.2]	10.7 [9.2, 12.5]	3.1 [2.3, 4.2]
Education									
No education	1,076	57.9 [53.6, 62.2]	42.1 [37.8, 46.4]	2 [1.2, 3.3]	5 [3.5, 7.2]	7.7 [5.9, 9.9]	25.7 [22.5, 29.3]	14.1 [11.6, 17]	3.5 [2.3, 5.1]
Primary	1,934	56.5 [53.5, 59.5]	43.5 [40.5, 46.5]	2.8 [2.1, 3.8]	6.6 [5.4, 8.1]	6 [5, 7.3]	23.2 [20.6, 26.1]	14.6 [12.8, 16.7]	3.2 [2.4, 4.2]
Secondary	3,219	43.2 [40.9, 45.5]	56.8 [54.5, 59.1]	3.5 [2.8, 4.4]	5.7 [4.6, 7.1]	4.7 [3.9, 5.7]	15.1 [13.4, 17.0]	11.2 [9.8, 12.7]	2.9 [2.3, 3.8]
Higher	736	31 [27.1, 35.2]	69 [64.8, 72.9]	5 [3.4, 7.3]	4.7 [3, 7.4]	3 [1.8, 5.1]	8.6 [6.4, 11.5]	8 [6.1, 10.5]	1.6 [0.9, 2.7]
Occupation									
Currently working	1,747	54.5 [51.4, 57.4]	45.5 [42.6, 48.6]	3.1 [2.2, 4.3]	5.8 [4.2, 8]	7.1 [5.8, 8.6]	21.9 [19.3, 24.7]	12.6 [10.8, 14.6]	4.0 [3, 5.2]
No	5,217	45.9 [44.1, 47.8]	54.1 [52.2, 55.9]	3.2 [2.7, 3.9]	5.7 [5, 6.6]	4.8 [4.2, 5.5]	17.3 [15.9, 18.8]	12.2 [11.1, 13.5]	2.6 [2.2, 3.2]
BMI, *n =* 6,946									
Underweight	1,556	58.2 [54.8, 61.5]	41.8 [38.5, 45.2]	2.6 [1.7, 4]	7.6 [6.1, 9.5]	8.6 [7.2, 10.4]	24.8 [21.8, 28]	10.1 [8.4, 12]	4.4 [3.4, 5.8]
Normal weight	4,067	49.3 [47.2, 51.4]	50.7 [48.6, 52.8]	3.6 [3, 4.3]	5.7 [4.7, 6.9]	5.2 [4.4, 6.1]	18.4 [16.9, 20.1]	13.8 [12.4, 15.3]	2.6 [2.1, 3.2]
Overweight and obesity	1,323	32.3 [29.1, 35.7]	67.7 [64.3, 70.9]	2.7 [1.8, 4]	3.6 [2.5, 5.2]	2.1 [1.5, 3.1]	11.1 [8.7, 14.1]	10.2 [8.5, 12.2]	2.5 [1.6, 3.9]
Wealth index									
Poorest	1,515	61.4 [57.8, 64.8]	38.6 [35.2, 42.2]	2.2 [1.4, 3.3]	6.7 [5.2, 8.6]	8.2 [6.8, 10]	27.4 [24.4, 30.7]	13.7 [11.7, 16.1]	3.1 [2.2, 4.3]
Poorer	1,307	55.5 [52, 58.9]	44.5 [41.1, 48]	3.6 [2.5, 5.1]	6.1 [4.7, 7.7]	7 [5.6, 8.8]	22.1 [19.1, 25.4]	13.2 [11.1, 15.7]	3.5 [2.5, 4.9]
Middle	1,379	48.1 [44.2, 52.1]	51.9 [47.9, 55.8]	3.3 [2.4, 4.5]	5.2 [4, 6.8]	4.1 [3.1, 5.5]	19.2 [16.1, 22.8]	12.9 [10.5, 15.8]	3.4 [2.4, 4.8]
Richer	1,420	42.8 [39.5, 46.1]	57.2 [53.9, 60.5]	2.9 [2.1, 4]	6 [4, 8.8]	4.4 [3.3, 5.8]	14.4 [12.2, 16.8]	12.5 [10.6, 14.8]	2.6 [1.9, 3.7]
Richest	1,344	30.6 [27.7, 33.7]	69.4 [66.3, 72.3]	4.3 [3.1, 6]	4.7 [3.4, 6.4]	2.8 [1.9, 4.2]	7.8 [6.3, 9.6]	8.9 [7.3, 10.8]	2.2 [1.3, 3.5]
Place of residence									
Urban	2,188	41.1 [38.5, 43.8]	58.9 [56.2, 61.5]	3.0 [2.2, 4.1]	4.7 [3.6, 6.1]	4.5 [3.5, 5.8]	14.4 [12.7, 16.4]	11.8 [10.2, 13.6]	2.6 [1.8, 3.7]
Rural	4,777	50.6 [48.7, 52.5]	49.4 [47.5, 51.3]	3.3 [2.7, 3.9]	6.1 [5.2, 7.1]	5.7 [5, 6.5]	19.9 [18.3, 21.5]	12.5 [11.3, 13.8]	3.1 [2.6, 3.7]
Exposed with media									
TV/Radio/Newspaper	4,315	43.4 [41.5, 45.4]	56.6 [54.6, 58.5]	3.3 [2.8, 4]	5.6 [4.7, 6.7]	4.3 [3.7, 5.1]	15.4 [14, 16.9]	12 [10.8, 13.4]	2.7 [2.1, 3.3]
Not at all	2,650	55.8 [53.1, 58.5]	44.2 [41.5, 46.9]	3 [2.3, 3.9]	6 [4.9, 7.2]	7.1 [6, 8.3]	23.5 [21.2, 25.9]	12.8 [11.3, 14.5]	3.5 [2.7, 4.4]

Abbreviations: ANC, antenatal care; ARI, acute respiratory infection; BMI, body mass index; CIAF, composite index of anthropometric failure.

Last 2 weeks.

* *P* <0.05.

** *P*< 0.001.

### 3.3 Geographical variations of CIAF

**Figure 1:**
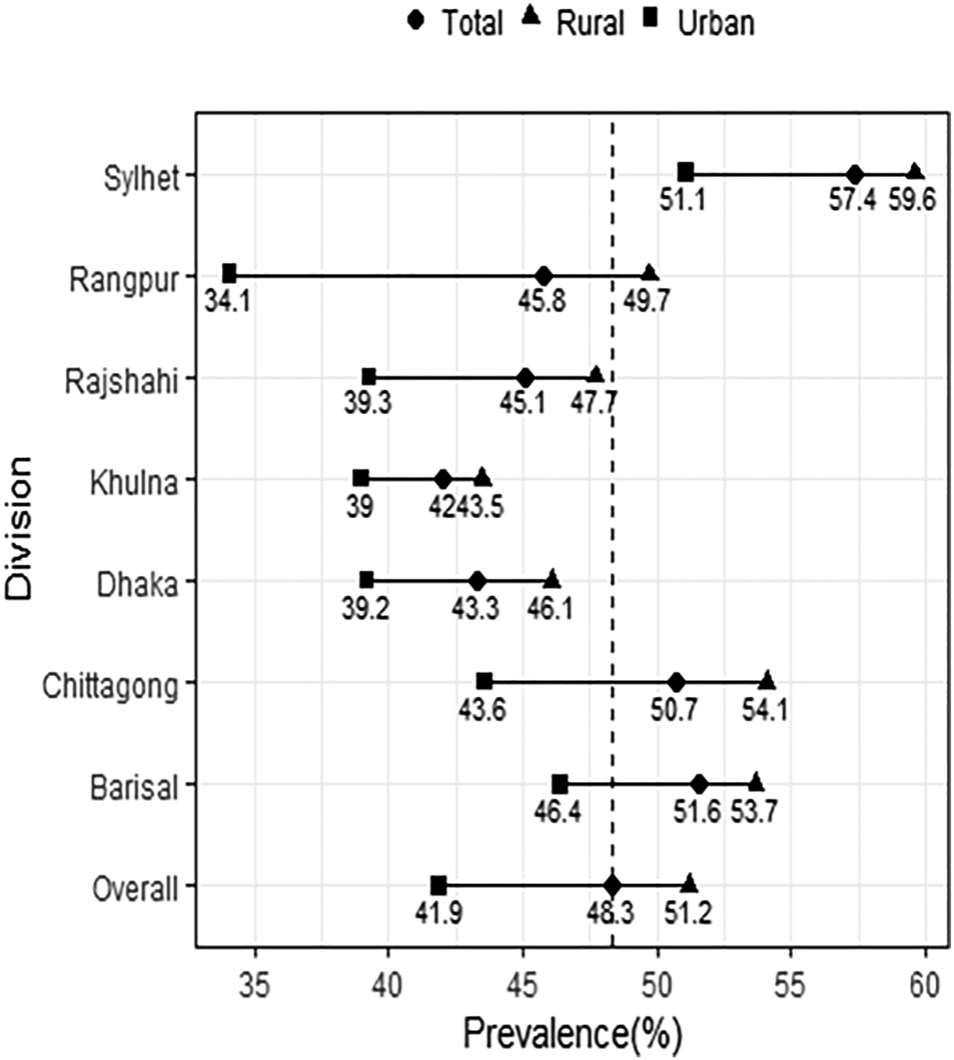
Prevalence of composite index of anthropometric failure by area of residence across seven administrative divisions

The prevalence of CIAF was also higher when the children live in rural areas compared with urban areas (51% vs. 41%; *P* < 0.001). The prevalence of CIAF was the highest in the Sylhet division (57%) and lowest in the Khulna division (42.0%). The prevalence of CIAF was higher in rural areas than in urban areas across seven administrative divisions (Figure 1). Similarly, the prevalence of all the category of CIAF was higher in the rural than its urban counterpart (Figure S2).

### 3.4 Factors associated with CIAF

In the bivariate analysis, the odds of CIAF were more likely among older age children (24–59 months), not to vaccinate their child, mode of birth is normal compared with C‐section, shorter preceding birth interval, lower order of birth of the index child, young maternal age at first birth (15–19 years), low education, currently working, lower socio‐economic position, living in rural areas, never exposed with media, mother received less than four ANC visit, size of child at birth was average or larger, had fever before 2 weeks of the survey, and undernutrition of mothers (Table 4).

**Table 4:** Factor associated with the Composite Index of Anthropometric Failure

	Crude OR* [95% CI]	*P* value	Adjusted OR *[95% CI]	*P* value
Sex of child				
Male	1.09 [0.96, 1.23]	0.084	,	
Female	1			
Child age				
24–59 months	1.42 [1.25, 1.61]	*P* < .001**	0.58 [0.33, 1.03]	0.064
0–23 months	1		1	
Mother age at first birth				
<20 years	1.39 [1.21, 1.6]	*P* < .001**	1.63 [1.03, 2.59]	0.039*
≥20 years	1		1	
Mother education				
No education	3.06 [2.3, 4.08]	*P* < .001**	1.30 [0.54, 3.12]	0.559
Primary	2.89 [2.36, 3.53]	*P* < .001**	1.07 [0.47, 2.43]	0.865
Secondary	1.69 [1.39, 2.05]	*P* < .001**	0.53 [0.27, 1.04]	0.066
Higher	1		1	
Occu*P*ation				
Currently working	1.41 [1.21, 1.64]	*P* < .001**	1.3 [0.71, 2.37]	0.400
No	1		1	
Wealth index				
Poorest	3.6 [2.84, 4.56]	*P* < .001**	3.29 [1.41, 7.67]	0.006*
Poorer	2.82 [2.33, 3.42]	*P* < .001**	2.04 [0.89, 4.71]	0.094
Middle	2.1 [1.72, 2.57]	*P* < .001**	1.24 [0.59, 2.58]	0.570
Richer	1.69 [1.37, 2.1]	*P* < .001**	0.84 [0.42, 1.71]	0.635
Richest	1		1	
Place of residence				
Rural	1.47 [1.23, 1.75]	*P* < .001**	0.47 [0.27, 0.84]	0.010*
Urban	1		1	
Media				
TV/Radio/Newspaper	1		1	
Not at all	1.65 [1.42, 1.92]	*P* < .001**	0.71 [0.41, 1.22]	0.214
Ever had vaccination				
Yes	1		1	
No	1.44 [1.02, 2.05]	.040	1.95 [1.12, 3.38]	0.019*
Delivery by C‐section				
Yes	1		1	
No	1.94 [1.58, 2.37]	*P* < .001**	1.62 [0.9, 2.92]	0.107
Preceding birth interval				
First birth	0.89 [0.77, 1.03]	.133	1.08 [0.63, 1.84]	0.784
<24 months	1.68 [1.3, 2.16]	*P* < .001**	1.39 [0.57, 3.42]	0.468
24–47 months	1.45 [1.25, 1.67]	*P* < .001**	1.16 [0.66, 2.01]	0.609
≥48 months	1		1	
Birth order				
1st	2.15 [1.75, 2.65]	*P* < .001**	4.31 [1.83, 10.11]	0.001*
2nd	1.8 [1.45, 2.24]	*P* < .001**	4.44 [2.39, 8.26]	*P* < .001*
3rd	1.61 [1.30, 2.01]	*P* < .001**	2.66 [1.54, 4.6]	0.001*
≥4	1		1	
Received ANC care				
≥4	1			
<4	1.75 [1.46, 2.11]	*P* < .001**	1.61 [0.97, 2.69]	0.068
Size of child at birth				
Average or larger	1.32 [1.09, 1.61]	.005*	1.67 [1.01, 2.76]	0.046*
Smaller than average	1		1	
Diarrhoea* (ref: no)	1.24 [0.94, 1.64]			
Fever^a^ (ref: no)	1.32 [1.18, 1.48]		0.89 [0.60, 1.32]	0.575
ARI (ref: no)	1.3 [0.94, 1.81]			
Mother body mass index				
Undernutrition	1.43 [1.2, 1.71]	*P* < .001**	1.60 [0.96, 2.68]	0.101
Overweight/Obesity	0.49 [0.4, 0.6]	*P* < .001**	0.77 [0.42, 1.42]	0.346
Normal weight	1		1	

Abbreviations: ANC, antenatal care; ARI, acute respiratory infection; 1OR: odds ratio.

* *P*<0.05.

** *P* < 0.001.

After adjusting all the covariates in the multivariable logistic regression model, we found that the odds of CIAF were more likely among young maternal age at first birth (OR: 1.63; 95% CI [1.03, 2.59]), poorest socio‐economic status (OR: 3.29, 95% CI [1.41, 7.67]), those mother who did not vaccinate their child (OR: 1.95, 95% CI [1.12, 3.38]), lower order of birth including first order (OR: 4.31, 95% CI [1.83, 10.11]), second order (OR: 4.44, 95% CI [2.39, 8.26]), third order (OR: 2.66, 95% CI [1.54, 4.60]), and average or larger size of child at birth (OR: 1.67, 95% CI [1.01, 2.76]). Our result showed that the odds of CIAF were less likely among children living in rural areas compared with urban areas (OR: 0.47, 95% CI [0.27, 0.84]; Table 4).

## 4 DISCUSSION

This study has applied the CIAF scale for estimating the overall burden of child under nutrition and identifying covariates. In Bangladesh, at least one in every two children under 5 years old has undernutrition, and one out of three children has both underweight and stunting. One community‐based study conducted in Bangladesh reported that 48% of rural and 58% of urban area children have undernutrition (Khan & Raza, 2014). This prevalence of CIAF was higher in many developing countries including India (Boregowda, Soni, Jain, & Agrawal, 2015; Dasgupta et al., 2015), Ethiopia (Endris, Asefa, & Dube, 2017), and Nepal (Goswami, 2016) and lower in Tanzania, Zimbabwe, Bolivia, and Peru (Nandy & Miranda, 2008) than the estimate of current study. This study revealed that the undernutrition status was higher among the children when they live in rural settings, if they are in the poorest socio‐economic position, if they did not receive any vaccinations, and if they are the firstborn. The high rate of child undernutrition may impact on the higher burden of morbidity due to lower immunization, which results in higher rates of mortality among the affected children (Ahmed et al., 2012).

We did not find any gender differences for overall undernutrition; however, in terms of stunting, only the proportion was higher for boys than girls. Overall undernutrition was significantly lower among younger age children, particularly among males. The children under 5 years old are at high risk for developing short‐ and long‐term consequences, irrespective of any gender differences. A meta‐analysis conducted in sub‐Saharan Africa reported that males are more stunted than females, which suggest males are more vulnerable to health inequalities than females (Wamani, Åstrøm, Peterson, Tumwine, & Tylleskär, 2007). One community‐based study in Bangladesh has suggested that socio‐economic disparities in stunting have increased over time (Rabbani, Khan, Yusuf, & Adams, 2016).

We found that wasting and underweight status are most prevalent among older age children than younger age groups and all other types of anthropometric failures were lower in the first 11 months of the child's life. The burden of underweight was almost similar across all the age groups. Similarly, studies from Ethiopia (Zelellw, Gebreigziabher, Alene, Negatie, & Kasahune, 2013) and Burkina Faso (Erismann et al., 2017) have shown that the proportion of undernutrition is increased as the age of the children increased. From 12 to 59 months, children have much physical and mental growth, and this time, a healthy balanced diet can support the development of the child's brain, and it can provide necessary nutrients as required. A community‐based study has shown that there is a clear link between food insecurity and malnutrition. One out of four households have food insecurity access in Bangladesh. The children aged 6 to 59 months old are at heightened risk of undernutrition (Hasan, Ahmed, & Chowdhury, 2013). A study in Bangladesh suggests that dairy intake can be extremely beneficial for reducing the stunting among children and that it can increase child growth (Choudhury & Headey, 2018). The government of Bangladesh targets to reduce the burden of stunting up to 25% by the end of 2025. A comprehensive community‐based intervention programme is crucial when reducing the burden of undernutrition.

In this study, we found that the children who live in the rural areas and who have low socio‐economic status are at higher risk of undernutrition, as well as when the mothers of the children had lower education level. A globally conducted systematic review reported that the relative difference in CIAF prevalence between the poorest and richest quintile has decreased and the difference between the lowest and highest education category has slightly increased in the low‐ and middle‐income countries including Bangladesh (Vollmer, Harttgen, Kupka, & Subramanian, 2017). One national study in Bangladesh has found that children of mothers who completed secondary and higher education had less growth failure, suggesting the education level have protective effects against underweight and wasting among children under 5 years old.

This study demonstrated that the prevalence of CIAF was 20% lower among the children who got an expanded programme on immunization vaccine. The children's immune system can automatically buildup through the vaccination, which can positively impact the reduction of undernutrition. In Bangladesh, the national immunization coverage is nearly 90%, which suggests that a majority of the children under 5 are now the coverage of the expanded programme on immunization. On the contrary, the children who did not receive vaccines were mostly from the rural area, were having poorer socio‐economic position, and were not more exposed to media than the families of their counterparts. This finding is consistent with one study conducted in Bangladesh (Fuchs, Sultana, Ahmed, & Iqbal Hossain, 2014).

Our study has found that undernutrition was significantly higher among first‐order children compared with the subsequent orders, and the pattern of undernutrition was persistent among the children who had a joint condition of undernutrition and stunting. According to the BDHS 2011, child mortality has significant associations with unwanted birth and order of children (Rahman, 2015). The higher order children usually get less attention for postnatal care and getting out from the coverage of the full vaccination rapidly. The findings suggest that order of birth of the children has an independent effect on the child's undernutrition, despite the contribution of other demographic and maternal characteristics. In Bangladesh, one out of four households has food insecurity status, which may impact on the nutrition of the higher birth order children (Hasan et al., 2013).

This sample size of the study is country representative, and the estimate of undernutrition reflects the real burden of undernutrition among children under 5 years old in Bangladesh. The nutritional indicators stunting, wasting, and underweight are measured following the WHO child growth standard. However, this study has some limitations, such as that the study is designed for cross‐sectional analysis, so we cannot interpret the significant covariates as risk factors of undernutrition. The data of child size at birth were collected according to the recall of the mothers of children, and therefore, the reporting of low birthweight may be overestimated or underestimated.

## 5 CONCLUSIONS

The finding from this study, which provides an overall burden of undernutrition based on CIAF, suggests that one out of two children under 5 years old are at risk of undernutrition. The burden was higher among the children who lived in the rural areas, or having a poor socio‐economic position, a lower education status of parents, a higher order of birth or a history of no BCG vaccination. Findings suggest that proper intervention programmes with targeting specific population groups are crucial to reducing the burden of undernutrition for achieving the sustainable development goal in improved nutrition by 2030 of Bangladesh.

## CONFLICTS OF INTEREST

The authors declare that they have no conflicts of interest.

## CONTRIBUTIONS

MSI conceptualized the study, performed the main data analysis, and drafted the initial manuscript. TB participated in interpretation of the data and revising the manuscript. All authors contributed to the development and approved the final manuscript.

## Supporting information

Figure S1: Flow diagram of the study participants

Figure S2: Prevalence of different form of anthropometric failure by the area of residence across seven administrative divisions
