# Supplementary material for: Prevalence and correlates of the composite index of anthropometric failure among children under 5 years old in Bangladesh
Source: Matern Child Nutr. 2019 Dec 22;16(2):e12930. doi: 10.1111/mcn.12930 (PMC7083426; doi:10.1111/mcn.12930)

Supplementary figure-1

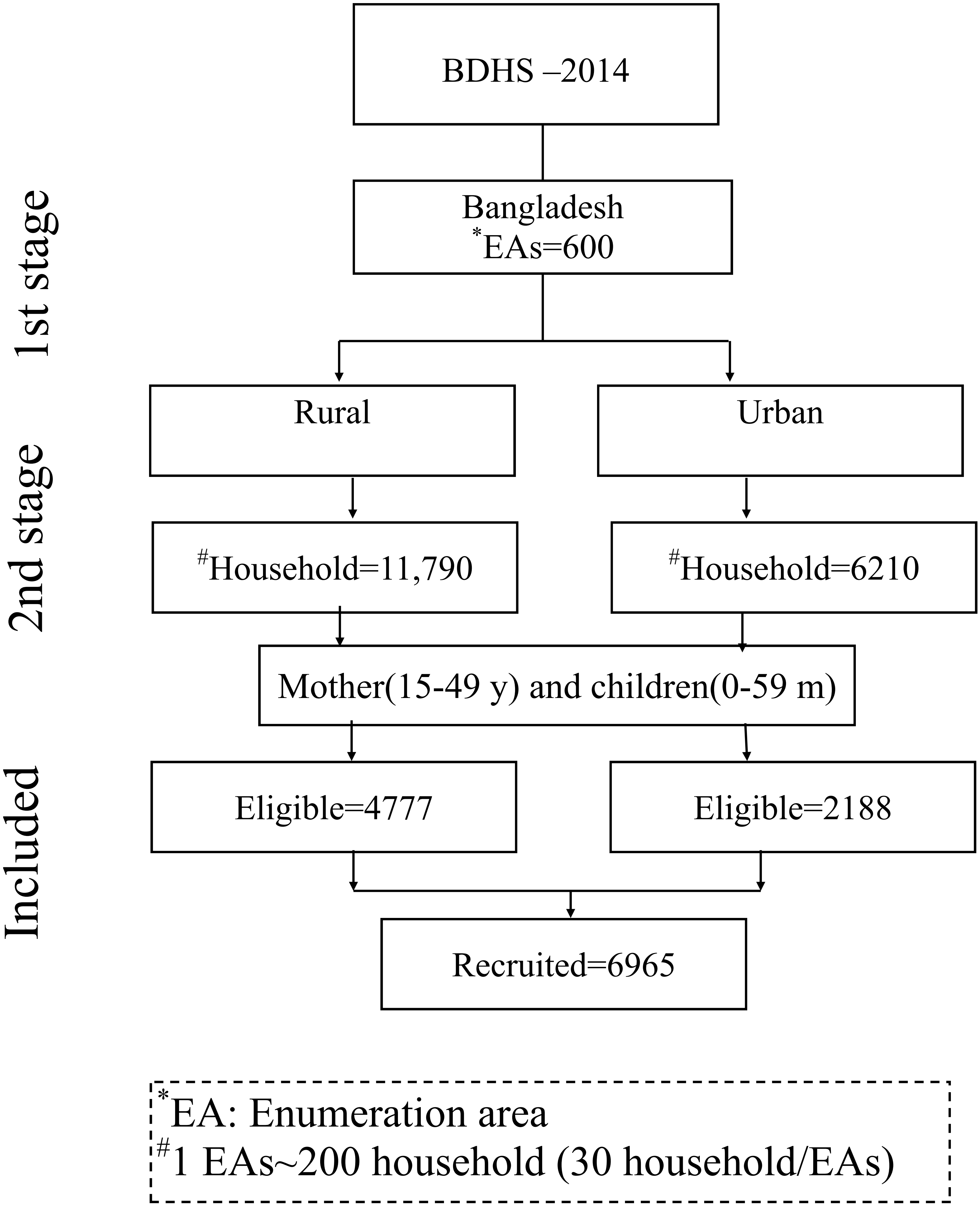

Supplement: Supplementary file 1 — Figure S1: Flow diagram of the study participants [file MCN-16-e12930-s001.pdf]
