# Supplementary figures and images for: Prevalence and correlates of the composite index of anthropometric failure among children under 5 years old in Bangladesh
Source: Matern Child Nutr. 2019 Dec 22;16(2):e12930. doi: 10.1111/mcn.12930 (PMC7083426; doi:10.1111/mcn.12930)

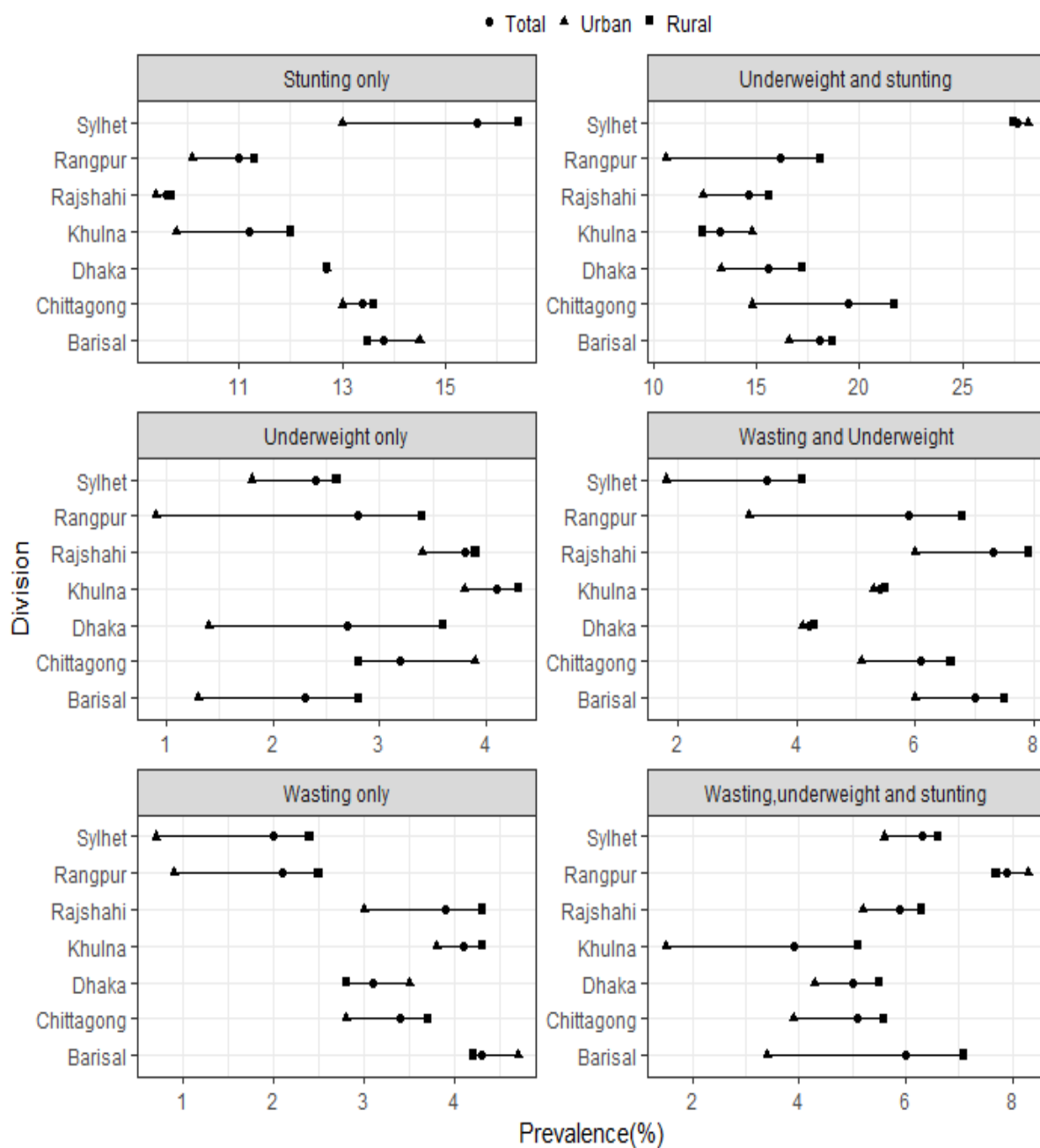

Supplement: Supplementary file 2 — Figure S2: Prevalence of different form of anthropometric failure by the area of residence across seven administrative divisions [file MCN-16-e12930-s002.pdf]
